# Detection of Patient HIV-1 Drug Resistance Mutations in Russia’s Northwestern Federal District in Patients with Treatment Failure

**DOI:** 10.3390/diagnostics12081821

**Published:** 2022-07-28

**Authors:** Alexander N. Shchemelev, Yulia V. Ostankova, Elena B. Zueva, Alexander V. Semenov, Areg A. Totolian

**Affiliations:** 1 Saint Petersburg Pasteur Institute, 197101 St. Petersburg, Russia; shenna1@yandex.ru (Y.V.O.); ezueva75@mail.ru (E.B.Z.); totolian@spbraaci.ru (A.A.T.); 2 Ekaterinburg Research Institute of Viral Infections of the Federal Research Institute, State Research Center for Virology and Biotechnology “Vector” of the Rospotrebnadzor, Rospotrebnadzor, 620030 Ekaterinburg, Russia; alexvsemenov@gmail.com

**Keywords:** human immunodeficiency virus, HIV, recombinant forms of HIV, HIV drug resistance, laboratory diagnostics

## Abstract

Highly active antiretroviral therapy (HAART) is currently a combination of three (less frequently four) antiretroviral drugs; these target pathways involved in various stages of HIV replication in the body. Treatment failure is a problem facing doctors and patients using HAART. The most common cause of therapeutic failure is the development of HIV drug resistance. The emergence of resistance is associated with processes involving mutation occurring in the viral genome under the influence of evolutionary factors. Sequencing reactions were performed using the AmpliSens HIV Resist-Seq. Assembly of consensus sequences from fragments obtained during sequencing was carried out using Unipro UGENE softwar. Isolate genotyping was performed using the MEGA-X software with the Neighbor-joining algorithm. According to the analysis, 72.05% of patients had at least one significant mutation associated with drug resistance for the corresponding viral subtype. HIV-1 A6 remains the predominant HIV-1 genetic variant in Russia’s Northwestern Federal District. Among samples with drug resistance mutations, in all cases, mutations associated with pharmacological resistance to two or three drug groups were found. Given the high incidence of resistance mutations in patients on ineffective ART, surveillance of HIV-1 drug resistance, in both ART-receiving and ART-naive individuals, appears necessary. A lack of vigilance and control measures may lead to the spread of primary ART-resistant HIV strains.

## 1. Introduction

The human immunodeficiency virus (HIV) is a retrovirus of the genus lentivirus that causes a slowly progressive disease upon infection [1]. Poorly controlled HIV infection can lead to a progressive, chronic syndrome characterized by the development of severe immunodeficiency, accompanied by a wide variety of concomitant and opportunistic illnesses. HIV infection belongs to the so-called ‘socially significant’ diseases that are dangerous for others; it is also recognized as a threat to national security [2].

The genetic diversity of HIV is a direct consequence of features of its rapid replication cycle. In the body of an infected individual, from 10^9^ to 10^10^ new viral particles are formed daily, while the mutation rate is from 10^−5^ to 10^−3^ errors/bp/cycle [3]. Given the fact that the size of the HIV genome is approximately 10,000 nucleotides, up to a million viral particles containing at least one mutation can be formed daily during uncontrolled infection. Another process contributing to HIV diversity is recombination, which involves exchange of RNA segments between different genetic variants of the virus. With coinfection, or superinfection (two or more HIV strains), recombination is almost inevitable [3,4].

Together, the processes of mutation and recombination in HIV evolution have led to the diversity currently seen. HIV-1 features three groups, designated as M, N, and O. Group M includes most of the common variants of the virus; the other two contain only a small number of strains [3,4]. Based on whole genome analysis, group M is further divided into 18 subtypes (denoted by letters A, B, C, D, etc.). Differences between subtypes are, on average, about 25–30% by genomic nucleotide sequence.

In terms of global HIV infections, subtype C (predominant in Asia and southern Africa) is the leader (47.2%), while in developed countries, subtype B is the most common (12.3%). The regional HIV epidemic (Russia and Commonwealth of Independent States countries) was caused by a subtype A variant, which continues to dominate in those territories [5,6]. With the exception of subtype B, all HIV-1 subtypes were formed on the African continent. Various aspects of human travel and mobility are thought to be behind the subsequent distribution unevenly around the globe [7].

Highly active antiretroviral therapy (HAART) is currently a combination of three (less frequently four) antiretroviral drugs; these target pathways involved in various stages of HIV replication in the body [8]. Treatment failure is a problem facing doctors and patients using HAART. The most common cause of therapeutic failure is the development of HIV drug resistance [9]. The emergence of resistance is associated with processes involving mutation occurring in the viral genome under the influence of evolutionary factors [9].

HAART has been in use for many years, and an ever-increasing number of patients are receiving it. As such, the emergence of transmissible drug resistance (DR) among HIV-infected individuals was a known, even expected, threat. Indeed, this outcome has already been seen in some countries [9]. To assess the level of primary resistance in a population of HIV-infected patients, the WHO publishes an updated list of DR mutations that should be taken into account during analysis [9,10]. In order to assess the resistance properties of strains that currently constitute a minority (10–20%) of a patient’s viral population, several factors must be considered: any previous viral resistance test results, features of HAART used, duration of drug administration, and any signs of treatment inefficacy [11].

To date, 25 antiretroviral drugs have been registered in Russia, which makes it possible to draw up more than 100 HAART regimens. These include two fusion inhibitors, eight nucleoside reverse transcriptase inhibitors, four non-nucleoside reverse transcriptase inhibitors, nine protease inhibitors, and two integrase inhibitors. According to the latest guidelines, TDF + 3TC + DTG and TDF + FTC + DTG are the preferred ART regimens, but EFV and two NRTI regimens remain the most common.

The local HIV epidemic began in the Soviet Union in the 1980s and is now rapidly developing in Russia. In the first half of 2020, 38,126 individuals with antibodies to HIV-1 were newly identified in Russia. By the end of the first half of 2020, 1,094,050 Russians with laboratory-diagnosed HIV infection were known to be living in the country [12].

In Russia, the dominant viral subtype is A6. In some publications, this subtype is referred to as: ‘IDU-A’, from the words ‘injecting drug users’, or ‘A-FSU’, from the words ‘former Soviet Union’ countries. Previously, this sub-subtype was classified as A1. However, due to significant differences between it and other HIV-1 subtype A1 variants in terms of structure and distribution, it was recategorized into a separate, relatively homogeneous group [9,13]. The aim of this work was to analyze the prevalence of HIV-1 drug resistance mutations in patients with ART failure in Russia’s Northwestern Federal District.

## 2. Materials and Methods

The study was approved by the Ethics Committee of the Saint Petersburg Pasteur Institute. It included analysis of HIV isolates obtained from 643 patients who contacted the Northwestern Federal District AIDS Center for diagnostic clarification of drug resistance status in the period 2014–2018. The inclusion criteria were as follows: those over 18 years of age, viral load above 1000 copies/mL, and no interruption in ART. The exclusion criteria were as follows: age less than 18 years, viral load below 1000 copies/mL, and interruption in ART (or no ART).

Quantitative analysis of HIV RNA was carried out with the AmpliSens^®^ HIV-Monitor-FRT commercial kit (Central Research Institute of Epidemiology, Russia), with a sensitivity threshold of 500 copies/mL. Samples with a detectable viral load (VL) were analyzed using RT-PCR and Sanger sequencing. For reverse transcription and amplification of HIV RNA, the RT-PCR-kit-Pro/Rev and PCR-kit-Pro/Rev commercial kits (Central Research Institute of Epidemiology, Russia) were used. Sequencing reactions were performed using the AmpliSens^®^ HIVResist-Seq kit (Central Research Institute of Epidemiology, Moscow, Russia) according to manufacturer instructions, as described earlier [14]. Sequencing was carried out using Applied Biosystems 3500 genetic analyzers according to the instructions.

Assembly of consensus sequences from fragments obtained during sequencing was carried out using Unipro UGENE software [15,16,17]. The consensus sequence included a 1302 nt region of the polymerase (pol) gene-encoding protease (PR) and a part of reverse transcriptase (RT/OT) in the 2253–3554 nt region; coordinates are given for HIV HXB2 in the GenBank database (K03455.1). The resulting sequences were analyzed for the presence of drug resistance mutations using the Stanford database [18]. Isolate genotyping was performed using the REGA HIV-1 Subtyping Tool 3.0 [19]. At the same time, analysis of phylogenetic relationships (between the genetic sequences of the studied strains and reference sequences from GenBank (Appendix A)) was carried out using MEGA-X software with the Neighbor-joining algorithm, which makes it possible to optimize trees in accordance with the “balanced minimum evolution” criterion. When assessing the reliability of phylogenetic relationships, we used multiple generations of samples using the bootstrap method for 1000 independent constructions of each phylogenetic tree [20]. Confidence intervals were determined by the Klopper–Pearson method.

## 3. Results

Of the 638 patients, more than half (62.54%) were male. The study group was dominated by the age category 18–34 years old (55.64%); the median age was 36 years. The ART regimen typically included two nucleoside reverse transcriptase inhibitors (NRTIs) plus one non-nucleoside reverse transcriptase inhibitor (NNRTI) (84.22%). Schemes with PI in the composition were much less common (15.78%). The most common schemes were TDF + 3TC + EFV and ABC + 3TC + EFV.

In 533 patients, an HIV-1 viral load greater than 1000 copies/mL was detected, which made it possible to obtain viral genome sequences encoding protease and reverse transcriptase. Some of the obtained and analyzed nucleotide sequences of the HIV-1 pol gene region were deposited in the international GenBank database under the numbers: MK510016-MK510079, MN317576-MN317587, OL505461-OL505538, ON367567-ON367728, and ON653444-ON653592.

**Figure 1:**
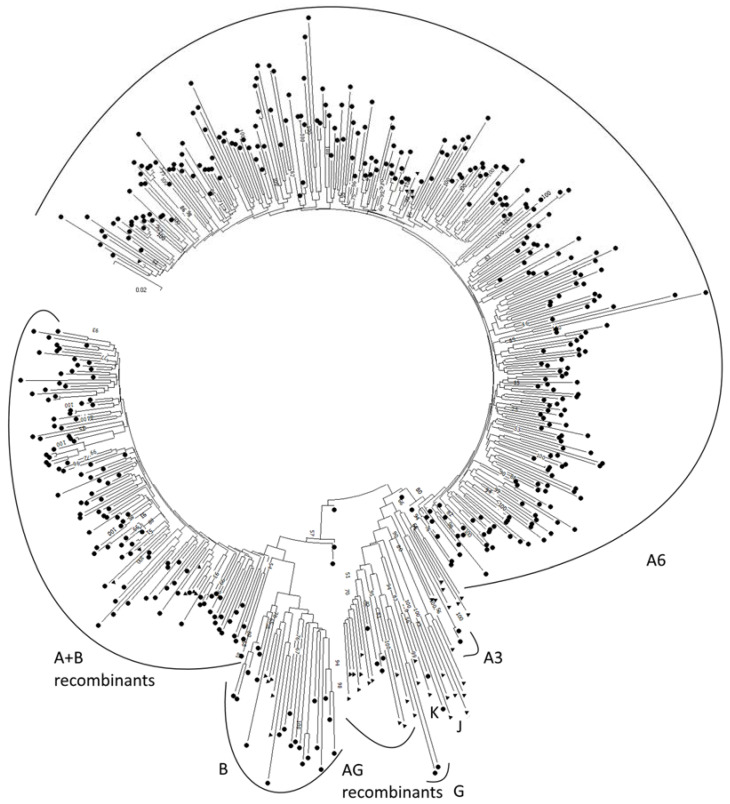
Result of phylogenetic analysis using Mega X. Key: ▲ reference sequences from GenBank; ● sequences of isolates from this study. Numbers on the nodes are bootstrap support values.

Two typing methods, phylogenetic analysis (Figure 1) and sequence analysis using the REGA HIV-1 Subtyping Tool 3.0, made it possible to more accurately assess the distribution of HIV-1 subtypes (Table 1).

According to the analysis, 72.05% of patients had at least one significant mutation associated with drug resistance for the corresponding viral subtype. In total, we encountered 140 different drug resistance mutations (75 NRTI, 65 NNRTI). The most common mutations in patients are presented in Table 2.

**Figure 2:**
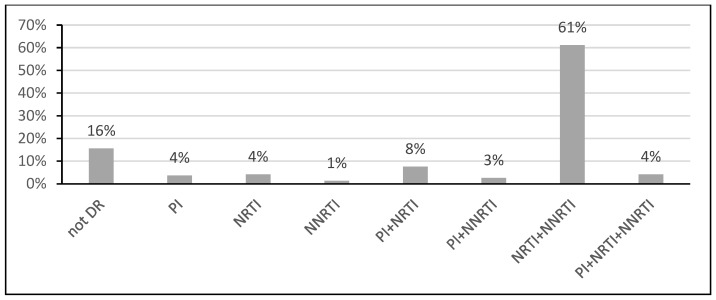
Prevalence of resistance to different drug classes in the study population. DR—drug resistance; PI—protease inhibitors; NRTI—nucleoside reverse transcriptase inhibitors; NNRTI—non-nucleoside reverse transcriptase inhibitors.

In the vast majority of studied isolates (60.98%), DR mutations (DRM) for NRTI + NNRTI drugs were encountered. In 3.94% of cases, multiple drug resistance (MDR) to three classes of drugs were found together (Figure 2).

Analysis of stable mutation combinations in the studied isolates showed thymidine analogue resistance mutation (TAM) patterns: TAM-1 (3.94% CI 2.46–5.96%) and TAM-2 (2.44% CI 1.30–4.13%). A stable non-TAM mutation combination was also seen: L74V + Y115F (8.82% CI 6.55–11.55%). In addition, stable combinations of mutations associated with DR to NNRTIs were identified: K101E + G190S (17.82% CI 14.67–21.34%), K103N (18.20% CI 15.01–21.74%), and K103N + V108I (8.26% CI 6.06–10.92%).

## 4. Discussion

The HIV-1 genetic diversity in the examined group matches known features of the situation in the Russian Federation: an absolute predominance of sub-subtype A6 (72%) [13]. It is important to note that when genotyping with the REGA HIV-1 Subtyping Tool 3.0, all isolates were assigned to sub-subtype A1. However, our own phylogenetic analysis allows us to assign them to sub-subtype A6 with full confidence. This discrepancy can be explained by the fact that the latest versions of the software used do not take into account data confirming the need to distinguish the A6 sub-subtype separately from the A1 sub-subtype. The next most common strains were recombinant forms between subtypes A and B (23%). This is due to the high prevalence of these recombinant forms in the Northwestern Federal District’s Kaliningrad region [6].

Genotyping is extremely important for testing of a sample for the presence of ARV-resistant HIV variants since viral genetic variants can differ in their biological properties, in the rate of viral evolution, and in disease progression, as well as in the contributions of various mutations to the formation of ART resistance. In this regard, additional studies are needed to assess the contribution of recombinant forms to the viral genetic diversity in the region. Insufficient attention to the high diversity of HIV recombinants, and a lack of complete data on common recombination points, can lead to an erroneous determination of the presence or absence of DR in the virus [21].

In addition, in a single case, a complex recombinant between CRF_03AB and the A1 subtype was encountered; our recombination analysis for it was carried out using the pol gene. The incidence of DR mutations in patients with virological failure of ART was extremely high. At the same time, mutations to reverse transcriptase inhibitors were most often found. Mutations associated with resistance to protease inhibitors were found in only eight cases (6%). This may be due to a higher genetic barrier to resistance with PIs, or their less frequent use in treatment regimens of the patients examined [9,22].

In comparison with 2012 data for St. Petersburg, the frequency of HIV DR mutations more than doubled from 30% to 72.05% [23]. At the same time, drug resistance in ART-naïve patients occurs in 5.5% of cases according to the Federal AIDS Center [13]. Such an increase in the number of resistant viral variants can be explained by changes in the population of HIV-infected people in Russia. During this period, the epidemic expanded beyond vulnerable groups of the population, and as a result, the social status of people living with HIV increased. Adherence generally increased, but still there were many patients and subgroups with suboptimal adherence, and such groups continue to pose a serious threat to the overall risk of DR development [24]. At the same time, the occurrence of transmissible primary resistance is increasing in the region; this undoubtedly contributes to the prevalence of drug resistance among people taking ARD.

Despite the increase in DRM prevalence, the ranking structure of mutations remained similar. The first place, in terms of occurrence, is still occupied by M184V (66.98%). In the second and third places were the G190S (30.96%) and K103N (23.08%) mutations. They are associated with simultaneous DR to several non-nucleoside reverse transcriptase inhibitors. A similar situation was observed in a 2012 study [23]. NRTI and NNRTI resistance mutations have occurred at similar rates, in many cases together, causing resistance to most reverse transcriptase inhibitors. A similar pattern of HIV DR mutations is found both in patients with primary drug resistance in Russia and in patients with newly diagnosed HIV and ART failure in some neighboring countries [13,25,26].

A dependence of substitution at position 190, featuring alanine (A) or serine (S), on the viral subtype was also revealed. The 190A substitution occurred only in strains of non-A subtypes, while the 190S mutation occurred mainly in strains of the A6 subtype. The literature also describes the prevalence of substitution at position 190 of reverse transcriptase with serine for subtype A [27] and alanine for non-A subtypes [28,29]

Mutations that are present in the viral genome at the moment of infection persist longer than those that arise during treatment. The duration of mutation persistence, to a certain extent, depends on its degree of influence on the replicative ability of the virus. For example, the M184V mutation reduces viral replication and is usually undetectable 5–20 weeks after HAART is discontinued. The K103N mutation can be detected 9–12 months after discontinuation of therapy or even later [11].

Combinations of mutations associated with resistance to thymidine analogs (TAM) described in detail in the literature were found in the obtained profiles in isolated cases, while patterns along the TAM-1 and TAM-2 trajectories were encountered with the same frequency. It is interesting to note that both patterns are associated with the T215Y mutation, yet it is known that patterns following the TAM-2 trajectory (with T215F) have an advantage [30]. Substitutions at 215 were seen in the studied mutation profiles, but not as part of standard TAM patterns.

Among PI drug resistance mutations, the major mutation M46I/L was seen in all cases, and the minor mutation L89T was seen in three cases. In addition, two mutations were identified in the tenth position of the protease region. One of them, L10LF, was identified in a single case; it is a minor PI resistance mutation. The other, L10I, was found in 40 cases (7.5%); it increases replication in viruses with other PI resistance mutations [31].

When analyzing the results of the study, it is also necessary to take into account the fact that standard genotyping assays require high minimum viral loads (1000 copies/mL) and detect only resistant variants making up more than 20% of the sample’s viral population. Perhaps the use of next-generation sequencing would allow for better identification of resistant HIV variants [32].

## 5. Conclusions

HIV-1 A6 (IDU-A) remains the predominant HIV-1 genetic variant in Russia’s Northwestern Federal District among patients with ineffective ART. A significant increase in the frequency of occurrence of HIV-1 drug resistance mutations in the region, compared to 2012, was shown. Among samples with drug resistance mutations, in all cases, mutations associated with pharmacological resistance to two or three drug groups were found. Given the high incidence of resistance mutations in patients on ineffective ART, surveillance of HIV-1 drug resistance, in both ART-receiving and ART-naive individuals, appears necessary. A lack of vigilance and control measures may lead to the spread of primary ART-resistant HIV strains.

## Figures and Tables

**Table 1:** Occurrence of HIV-1 subtypes in the studied isolates.

Subtype	Number of Isolates	Percent	95% Confidence Interval
A6	383	71.86%	67.83–75.64%
A3	1	0.19%	0–1.04%
B	21	3.94%	2.46–5.96%
G	2	0.38%	0.05–1.35%
K	1	0.19%	0–1.04%
J	1	0.19%	0–1.04%
CRF02_AG	4	0.75%	0.20–1.91%
CRF03_AB	70	13.13%	10.38–16.30%
CRF03_AB + A	51	9.57%	7.21–12.39%

**Table 2:** Most common drug resistance mutations.

Mutation	Number of Isolates	Percent	95% Confidence Interval
NRTI mutations			
M184V	357	66.98%	62.81–70.96%
L74V	85	18.95%	12.94–19.34%
A62V	80	15.01%	12.08–18.33%
K65R	75	14.07%	11.23–17.32%
D67N	54	10.13%	7.70–13.01%
**NNRTI mutations**			
G190S	165	30.96%	27.05–35.07%
K103N	123	23.08%	18.56–26.89%
K101E	117	21.95%	18.51–25.71%
Y181C	74	13.88%	11.06–17.11%

## Data Availability

Data are available on request from the authors.

## Appendix A

**Table A1:** Complete sample names of reference sequences.

Complete Sample Name	Subtype in REGA (Version 3.0)	Subtype by Phylogenetic Analysis
AB098332	A3	A3
AB231896	CRF 02AG	CRF 02AG
AB231898	CRF 02AG	CRF 02AG
AB287376	A1	A1
AF061641	G	G
AF063224	CRF 02AG	CRF 02AG
AF067155	C	C
AF069670	A1	A1
AF075703	F1	F1
AF084936	G	G
AF107771	A	A
AF286237	A2	A2
AF377954	CRF 02AG	CRF 02AG
AF413987	A6	A1
AF484509	A1	A1
AY151001	CRF 02AG	CRF 02AG
AY173951	B	B
AY500393	A6	A6
AY521629	A3	A3
AY521631	A3	A3
AY713409	B	B
AY772699	C	C
EF589043	A6	A6
EU110087	A1	A1
EU786671	CRF 02AG	CRF 02AG
EU861977	A1	A1
GU201514	CRF 02AG	CRF 02AG
HM586190	B	B
HQ161930	A6	A6
HQ449397	A6	A6
KJ771697	B	B
KT124792	CRF 02AG	CRF 02AG
M17449	B	B
U46016	C	C
U51190	A1	A1
U52953	C	C
U88826	G	G
MH605500.1	CRF 06cpx	CRF 06cpx
HQ529257.1	CRF 06cpx	CRF 06cpx
